# Mastermind: A Comprehensive Genomic Association Search Engine for Empirical Evidence Curation and Genetic Variant Interpretation

**DOI:** 10.3389/fgene.2020.577152

**Published:** 2020-11-13

**Authors:** Lauren M. Chunn, Diane C. Nefcy, Rachel W. Scouten, Ryan P. Tarpey, Gurinder Chauhan, Megan S. Lim, Kojo S. J. Elenitoba-Johnson, Steven A. Schwartz, Mark J. Kiel

**Affiliations:** ^1^Genomenon Inc., Ann Arbor, MI, United States; ^2^The Johns Hopkins Hospital, Department of Pharmacy, Baltimore, MD, United States; ^3^Division of Hematopathology, Department of Pathology and Laboratory Medicine, University of Pennsylvania, Philadelphia, PA, United States; ^4^Division of Precision and Computational Diagnostics, Department of Pathology and Laboratory Medicine, University of Pennsylvania, Philadelphia, PA, United States

**Keywords:** genome sequence analysis, copy number variant, rare disease, oncology, gene fusions, variant interpretation and classification

## Abstract

Design and interpretation of genome sequencing assays in clinical diagnostics and research labs is complicated by an inability to identify information from the medical literature and related databases quickly, comprehensively and reproducibly. This challenge is compounded by the complexity and heterogeneity of nomenclatures used to describe diseases, genes and genetic variants. Mastermind is a widely-used bioinformatic platform of genomic associations that has indexed more than 7.5 M full-text articles and 2.5 M supplemental datasets. It has automatically identified, disambiguated and annotated >6.1 M genetic variants and identified >50 K disease-gene associations. Here, we describe how Mastermind improves the sensitivity and reproducibility of clinical variant interpretation and produces comprehensive genomic landscapes of genetic variants driving pharmaceutical research. We demonstrate an alarmingly high degree of heterogeneity across commercially available panels for hereditary cancer that is resolved by evidence from Mastermind. We further examined the sensitivity of Mastermind for variant interpretation by examining 108 clinically-encountered variants and comparing the results to alternate methods. Mastermind demonstrated a sensitivity of 98.4% compared to 4.4, 45.6, and 37.4% for alternatives PubMed, Google Scholar, and ClinVar, respectively, and a specificity of 98.5% compared to 45.1, 57.6, and 68.8% as well as an increase in content yield of 22.6-, 2.2-, and 2.6-fold. When curated for clinical significance, Mastermind identified more than 4.9-fold more pathogenic variants than ClinVar for representative genes. For structural variants, we compared Mastermind’s ability to sensitively identify evidence for 10 representative disease-causing CNVs versus results identified in PubMed, as well as its ability to identify evidence for fusion events compared to COSMIC. Mastermind demonstrated a 4.0- to 43.9-fold increase in references for specific CNVs compared to PubMed, as well as 5.4-fold more fusion genes when compared with COSMIC’s curated database. Additionally, Mastermind produced an 8.0-fold increase in reference citations for fusion events common to Mastermind and outside databases. Taken together, these results demonstrate the utility and superiority of Mastermind in terms of both sensitivity and specificity of automated results for clinical diagnostic variant interpretation for multiple genetic variant types and highlight the potential benefit in informing pharmaceutical research.

## Introduction

### Need for Improved Access to Information in the Medical Literature for Clinical Genomics

Next-generation DNA sequencing (NGS) has made it possible to sequence patient genomes at scale and to diagnose and treat a much broader and more diverse group of indications. This technique is also used extensively in research labs to uncover novel genomic associations with disease resulting in thousands of new articles on human genetic variants being added each week to the over 30 million existing medical articles listed in the National Library of Medicine/MEDLINE/PubMed database. The information in a single article can often mean the difference between an American College of Medical Genetics/Association of Molecular Pathology (ACMG/AMP) designation of variant of uncertain significance (VUS) and a variant deemed to be likely pathogenic and therefore clinically actionable. Missing even one article from among these millions can significantly impact the accuracy of clinical variant calling, reducing the chance that patients receive the best and most appropriate care. Therefore, having ready access to the most complete database of published variants and all the associated evidence annotations is essential in reducing the time it takes to interpret a variant and ensuring the accuracy of that interpretation. Moreover, an inability to interrogate the full breadth and depth of the empirical evidence in the scientific literature frustrates the efforts of geneticists and pharmaceutical researchers seeking to assemble the most comprehensive and thoroughly annotated dataset of these causative variants with clinical and functional annotations.

More specifically, limited access to information in the medical literature is one of the biggest bottlenecks preventing more automated and more reproducibly accurate diagnostic sequencing panel design and variant interpretation for diagnosis of genetic diseases like cancer and constitutional conditions based on these sequencing assays. This challenge is due to the inability to search through the entirety of this body of knowledge and also due to the complexity and heterogeneity of genetic variant nomenclatures in addition to the challenge of recognizing entities such as diseases, genes and drug compounds in these texts. Current solutions to collecting this information include manually searching through PubMed which only permits title and abstract searching or otherwise using Google Scholar which doesn’t reconcile disparate biological entity nomenclatures. Both of these widely used techniques therefore both suffer from a lack of sensitivity. Moreover, when searching using PubMed or Google Scholar, many false positive results are returned for instance, owing to the inability to properly determine to which gene any given variant cited in an article is referring leading to a decrease in specificity. Finally, laborious and error-prone manual review of this data is also a significant challenge even if all the results needed to make an informed conclusion are available.

Mastermind is a genomic search engine that allows users to search a comprehensive dataset comprising the medical literature, pre-annotated for genetic content, which is intended to resolve these challenges (Genomenon Inc., 2020a). In addition to the search engine user-interface, there are also two other useful tools for evaluation of the genomic associations identified by Mastermind including the Cited Variants Reference (CVR; Genomenon Inc., 2020b) and the Mastermind API (Genomenon Inc., 2020c). The CVR is a download of Mastermind’s database that includes all variants affecting less than 4 nucleotides along with the number of references in the standard VCF format. The Mastermind API also allows for more comprehensive access to Mastermind’s database through programmatic use of a number of endpoints including genes, variants, diseases, phenotypes, and therapies.

We demonstrate here, for multiple applications, the benefits of applying the automated genomic association indexing capability of Mastermind for solving several challenges facing clinical diagnostic genetics and pharmaceutical research driven by genomics.

### The Need for Evidence-Based Gene Panel Design

Next-generation DNA sequencing has fundamentally changed the way molecular diagnostic assays are designed and performed in clinical laboratories. One of the most consequential changes that has resulted is the trend toward larger and larger numbers of genes being sequenced per patient and the consolidation of use cases into single, so-called comprehensive gene panels due to the ease and cost-efficiency of producing this data using NGS. The genes selected for sequencing for a given indication are bundled into discrete units known as gene panels. This bundling into gene panels is helpful to streamline clinical laboratory workflows and assay validations as multiple disparate sequencing assays can be consolidated into a single assay. Moreover, larger gene panels afford the opportunity for labs to identify a wider variety of clinically meaningful biomarkers at a lower cost for any given patient.

Diagnostic gene panels are used in clinical practice to confirm diagnoses, to inform prognostic determinations, and increasingly to tailor therapy to molecular etiology of disease, particularly in oncology. Many commercial and academic reference laboratories offer sequencing as a service using pre-defined gene panels for many different clinical circumstances. In particular, many of these reference laboratories are offering larger and larger gene panels for a wide variety of diseases including gene panels intended to be used for multiple cancer types.

The selection of the most relevant genes as biomarkers on these NGS panels is typically performed using labor-intensive, manual consultation of available databases and medical literature followed by data organization in a process that can take many months to complete and is non-scalable. This process is often predicated on incomplete and subjective information gathered by individuals with widely differing levels of experience. Moreover, within the typical process used to select content for gene panels, the criteria utilized can be highly variable. Gene panel composition, for example, can be influenced by factors that require limiting the number of genes selected for the final assay, such as the inclusion or exclusion of known fusion genes or of genes with a desired level of certainty regarding their association with the given diagnostic target.

Because of the degree of subjectivity introduced by those criteria and inherent in the process overall, there is the potential for substantial discordance between commercial gene panels that purport to be useful for the same indications. In this study, we introduce an evidence-driven automated literature curation approach using Mastermind for identification and interpretation of genes for inclusion on such sequencing panels. To demonstrate the value of this approach, we sought to investigate the extent to which there is discrepancy among gene targets comprising multiple commercial panels. In response, we present an alternate, evidence-based approach to interrogating the medical literature using text-mining and automated bio-curation techniques to make this process more reproducible as well as reveal sufficient evidence for each biomarker candidate to allow for rapid confirmation and assessment of final panel design.

### The Need for More Complete Variant Databases to Foster Accurate and Reproducible Variant Interpretation Techniques

Analogous to the design and use of these panels, consistent analysis of the sequencing data that results for any given patient remains a significant issue for clinical laboratories. For the results of any sequencing assay to be clinically actionable (including those resulting from gene panels but also those resulting from exome or genome sequencing), each variant discovered in a particular patient must be accurately interpreted as being pathogenic, benign, or a VUS. This interpretation relies on consultation of a number of databases that serve different purposes in the variant interpretation process and are, at times, dependent on the type of variant and/or disease being investigated. These databases include population frequency databases [e.g., gnomAD (gnomAD, 2020) and 1,000 Genomes], databases for single nucleotide polymorphisms (e.g., dbSNP), databases for characterization of genetic diseases [e.g., OMIM (Online Mendelian Inheritance in Man [OMIM], 2020) and Orphanet (Orphanet, 2020)], databases for somatic variants in cancer (e.g., COSMIC and The Cancer Genome Atlas), databases for computational predictions of functional consequences [e.g., PolyPhen (PolyPhen-2, 2020), SIFT (Ng and Henikoff, 2001), and MutationTaster (Schwarz et al., 2010)], databases for structural variants (e.g., the Database of Genome Variants, DECIPHER, and CNVDigest), and more comprehensive variant databases [e.g., ClinVar (National Center for Biotechnology Information [NCBI], 2020b) – somatic, germline, and structural variants, HGMD (Cooper et al., 1998) – germline variants, AVADA (Birgmeier et al., 2020) – monogenic disease variants]. Importantly, however, the variant interpretation process additionally requires consultation of the medical literature and detailed examination of its contents, carried out largely by manual searches of public databases such as PubMed and Google Scholar – a process that can take up to 3 h per variant. This allows for substantial discordance across clinical laboratories (Hoskinson et al., 2017).

In 2015, the American College of Medical Genetics (ACMG) released a set of criteria for determining the pathogenicity of variants for Mendelian disorders in an attempt to provide more substantial guidelines that could remedy this discordance (Richards et al., 2015). However, this attempt appears to be only partially successful. One study found that for 9 clinical laboratories, there was only 34% concordance in variant interpretation that increased to 71% once the laboratories discussed all the prevailing evidence and further clarified the criteria (Amendola et al., 2016). Additionally, the concordance between these laboratories’ own criteria for variant interpretation and ACMG criteria was higher, at 79%, representing the subjectivity of applying these criteria. This study attests to the difficulty of ensuring consistent interpretation when criteria are not made sufficiently clear as well as when evidence is not provided systematically.

Paramount to resolving the heterogeneous results of interpretation across different labs is a need to ensure all interpreters begin with the same evidence with which to draw their final conclusions. Critical metrics to consider when assessing the comprehensiveness and quality of such a variant database include rates of both false negatives and false positives. Specifically, the ideal database will necessarily.

• Minimize false negatives by ensuring adequate searching of full-text and supplemental data.
• Minimize false negatives by ensuring the broadest coverage of empirical evidence.
• Minimize false negatives by ensuring a comprehensive variant indexing process.
• Minimize false positive variants by eliminating text matches that merely resemble variants.
• Minimize false positive variants by ensuring correct identification of corresponding genes.

Beyond matching standardized variant nomenclatures, search techniques must be able to recognize a number of non-standard nomenclatures, styles, and special characters that are used to describe variants in the published literature and even more so in the heterogeneous supplemental data. Capturing all the variant types and all the ways an author can describe a variant takes years of development and domain experience. The broad range of both variant types and nomenclatures exposes the indexing process to the increased likelihood of numerous false positives resulting from inappropriate variant matches and faulty gene-to-variant pairing.

Solving these challenges requires years of development and iterative improvement in eliminating these false positives in a systematic fashion and requires an ongoing commitment to improving search result quality with a specific focus on genetics and genomics. By implementing an automated approach to literature curation utilizing the entirety of the genetic literature as well as an organized and useful user-interface, we demonstrate the improved consistency and accuracy of variant interpretation resulting from automated indexing of the empirical evidence through Mastermind.

### The Need for More Complete Clinical-Grade Structural Variant Databases

CNVs – DNA segments of 1,000 base pairs or more that are either deleted or amplified in a single genome - are increasingly recognized as the cause of a variety of diseases, including cancer and hereditary constitutional diseases. They are causative due to the likelihood of altering gene expression levels due to a gene dosage effect. The complexity and heterogeneity of different CNVs and the difficulty in determining *a priori* what the pathogenic consequence of any given CNV may be (as it can influence many hundreds to thousands of genes on any one locus) make it challenging to find the information needed to properly ascribe pathogenicity. Nevertheless, vast amounts of CNV data are published in the historical and current literature that are critical to providing patients with an accurate diagnosis. A recent study suggested that 15–25% of clinical cases submitted for genome sequencing are caused by CNV driver variants. Moreover, current databases that contain CNV information lack the comprehensiveness required to be clinically useful. One such database, the Database of Genome Variants (DGV^1^) (The Center for Applied Genomics, 2020), is vast but contains polymorphic CNV data from healthy individuals. ClinGen^2^ (ClinGen - Clinical Genome Resource, 2020) seeks to catalog disease-gene associations but to date has only reviewed 1,778 genes across the many thousands known or perceived to be disease-causing due to their manual approach to database assembly. Moreover, ClinGen’s manual evidence review procedures make a comprehensive approach to identifying relevant information impossible. Another database, DECIPHER^3^ (DECIPHER, 2020) lacks the empirical evidence supporting each identified CNV as disease-causing limiting its utility for clinical purposes.

Two recent publications highlighted this need and proposed solutions that culminated in the Copy Number Variation in Disease (CNVD Database; Qiu et al., 2012) and the CNVdigest^4^ (CNVdigest, 2020). However, the CNVD was manually produced by examining fewer than 10,000 references and suffers from an inability to scale. On the other hand, the CNVdigest database utilizes an automated text-mining approach, but it only indexes the MEDLINE title and abstract data and PubMed Central full-text articles and is to date limited to fewer than 50,000 references. It is therefore missing a significant fraction of the empirical evidence in the medical literature including many millions of full-text articles and supplemental datasets. Finally, neither of these databases were available at the time of this writing as the websites returned server errors. Given this need, we sought to apply the ability of Mastermind to identify CNVs and compared these results to results obtained using standard PubMed searches.

In addition to CNVs, another class of larger structural alterations influences disease development, progression and response to therapy – fusion genes. For instance, fusion genes have long been known to play an important role in the development of cancer (Mitelman et al., 2007). Since the discovery of the first fusion gene in 1960, researchers have continued to discover fusion events involved in cancer development. In recent years, hundreds of novel fusions have been identified across a multitude of cancer types, due in large part to the ease of producing this data using next generation sequencing techniques like RNAseq and their broad use in clinical diagnostic labs. Fusion genes therefore are playing an increasingly important role in clinical diagnosis. Identifying and documenting each newly discovered fusion is crucial in both patient diagnosis and the development of precision medicine.

This diagnostic modality allows for the proper application of existing therapies and the development of new therapies. Two recent studies reported that 6–15% of patients with metastatic cancer harbored genomic rearrangements, many of which produced putative fusions (Zehir et al., 2017; Gao et al., 2018). While 35% of these fusions involved kinase genes, indicating they could possibly be targeted by kinase inhibitors, 19% of them involved novel partner genes. Currently, the Catalog of Somatic Mutations in Cancer (COSMIC) and OncoKb serve as the main source for documented fusions (Tomczak et al., 2015; Tate et al., 2019). At the time of this writing, COSMIC contains 297 unique fusion pairs derived from ∼1.4 million tumor samples, while OncoKb contains data from literature curation. We applied Mastermind to identify fusion events and compared the results to data contained in these two databases.

## Methods

### Extraction of Information From Empirical Evidence

All data discussed below was assembled by indexing the full-text and supplemental material of prioritized references identified in MEDLINE/PubMed^5^ (National Center for Biotechnology Information [NCBI], 2020a). These references were evaluated to identify diseases, genes and genetic variants (including single-nucleotide changes, indels, copy number variants, fusion genes and karyotypic abnormalities) and all supporting information (including functional and clinical key terms) as detailed in the Supplementary Material.

Titles and abstracts and additional data fields (including Substances, Genes, and MeSH fields) for all references available through MEDLINE (using the *eutils* API^6^) (Sayers, 2010) were indexed for the mention of diseases and genes using the ontologies described in each subsequent methods section. References were identified by PubMed identification number (PMID) and full-text and supplemental materials were indexed after prioritization based on the information content of the title. This prioritization was based on abstract and/or full-text indexing results; for instance, the mention of any one of the diseases, genes or supporting key terms in the title, abstract, or metadata fields. Data are indexed with a custom-built, multi-step process. A variety of common font-encoding and character set problems are corrected using custom designed data processing tools. Extraneous parts of the article such as references sections are also detected and excluded from processing using Grobid^7^ (Lopez, 2020). The text of the article was searched for diseases, phenotypes, therapies, genes, and keywords, using an extensive set of synonyms, ontologies, and downstream processes described below.

This information is publicly available through the Mastermind Genomic Search Engine^8^ (Genomenon Inc., 2020a) and in downloadable form^9^ (Genomenon Inc., 2020b). All information is referenced under the protection of the fair use provision of applicable copyright law. Mastermind users are encouraged to examine the full extent of published, copyrighted content available to them only through licensed access provided by the respective publishers or distribution channels such as PubMed. All appropriate citations are included with each annotated fact derived from these materials in the Mastermind software.

### Diseases and Genes

#### Disease and Gene Identification

Diseases and their synonyms were identified by Medical Subject Heading Terms (MeSH^10^) (National Center for Biotechnology Information [NCBI], 2020d) and gene symbols by HGNC nomenclature^11^ (Hugo Gene Nomenclature Committee [HGNC], 2020). Synonyms and aliases from a variety of additional sources including UniProt^12^ (UniProt, 2020) were incorporated and treated as equivalent to accepted names or symbols in the indexing process. Manual review of a summary of the complete output of this indexing process was performed to enhance the specificity of these results by removing highly non-specific synonyms.

#### Mastermind Hereditary Cancer Gene Panel

The Mastermind Hereditary Cancer Gene Panel was created for the 206 genes that were included on at least one of the 8 hereditary cancer panels that were evaluated. Per gene, articles that mentioned the gene within the title and/or abstract were identified and then prioritized first by the presence of the gene within the title, then by the citation count as a measure of relevance, and finally by the presence of one or more cancer-related keywords within the title. For the top 100 of these prioritized articles, the sentences from the full-text that contained the input gene were extracted from the Mastermind database and evaluated by the presence of keywords related to a particular genetic mechanism: mutation, copy number variation, fusion, or expression level change. This was represented as a percentage of the total sentences evaluated that contained a keyword related to the respective mechanism. The top 10 of the prioritized PMIDs and their titles were subsequently displayed in the final panel.

#### Commercial Disease-Gene Panel Comparison

Comprehensive gene panels available commercially were identified using a combination of Concert Genetics^13^ (Concert Genetics, 2020), the Genetic Testing Registry^14^ (National Center for Biotechnology Information [NCBI], 2020c) and Google searching^15^ (Google, 2020a) to mimic the typical process of initially identifying relevant clinical assays undertaken by clinical offices. The composition of each panel was ascertained from respective laboratory websites and organized into a spreadsheet (Supplementary Table 1). Gene names that did not conform to HGNC nomenclature were manually corrected and the overlap among each panel was determined. For examination of the evidence in the literature available for biomarkers not on a consensus of comprehensive hereditary gene panels, each gene was queried using Mastermind and the total number of abstracts and full text articles mentioning each gene as well as the aggregate number of mentions of that gene. For development of the automated hematological panels, each PMID containing the mention of the MeSH term “Leukemia” or “Lymphoma” was further assessed for the mention of any gene symbol or synonym and the data organized into disease-gene associations. A comprehensive itemization of the genes associated with leukemia/lymphoma was thereby listed in descending order of the number of references that cite each gene. For this analysis, any references mentioning the targeted gene were prioritized first by the gene’s presence in the article’s title, then the citation count of the article as a measure of relevance (in descending order), and finally the presence of cancer related keywords in the article’s title. Additionally, the genetic mechanism(s) referenced to the targeted gene were analyzed by retrieving the number of related keywords from sentences mentioning the gene from the top 100 prioritized articles as a percentage of the total number of sentences mentioning the gene. Enhancements to the Mastermind user interface that facilitate the automation of gene panel design are scheduled for release between 2020 and 2021 though all the results of our analysis can currently be reproduced manually using Mastermind as described above. Alternatively, motivated users can collect the requisite data and associated reference citations using the Mastermind API^16^ which offers a variety of endpoints to repeat our analyses in high throughput.

### Single-Nucleotide and Indel Variants and Variant Interpretation

#### Single-Nucleotide and Indel Variant Identification

Identification of single-nucleotide and indel variants relies on recognition of variant forms in the text using numerous regular expressions. The results of these matches are reconciled to cDNA or protein positions as appropriate or otherwise matched to rsIDs found in dbSNP^17^ (National Center for Biotechnology Information [NCBI], 2020e). Each variant is then mapped to a Mastermind database key based on its protein-level effects. The article text and the set of matched genes are then indexed for variants by indexing article text using a finite state machine that recognizes variant descriptions in standard HGVS notation, by rsID, and in other less commonly-used forms, e.g., ‘T1799A’ for cDNA variants, ‘ΔR30,’ and so on. Matches are evaluated in the context of a set of candidate genes for that paper, with each variant being checked for validity against transcript and protein sequences for each gene. cDNA variants are converted to normalized protein-level representations, associated with genes, converted to normalized Human Genome Variation Society (HGVS^18^) (Human Genome Variation Society [HGVS], 2020) representations, and assigned Mastermind key values. Finally, appropriate gene-variant associations are selected in a whole-article context after considering numerous textual relationships and then stored in Mastermind. When generating Mastermind keys for matches, cDNA positions are converted to single-letter amino acid positions, e.g., V600E. For intronic variants, the amino acid coded for at the 5′ side of the intron is used, so *BRAF* would have E80sd, E80int, and E80sa. Splice sites are the first and last two bases of the intron. Splice regions include additional nucleotides in the intron as well as coding variants flanking the exon-intron and intron-exon junctions and are designated as srd and sra, respectively. Similarly, for frameshift variants, the index amino acid is used and the Mastermind key designated with an fs, as in M123fs. Variants in the 5′ and 3′ UTR regions are similarly grouped together regardless of cDNA change and assigned Mastermind keys of 5UTR and 3UTR. In the user interface of the Mastermind Genomic Search Engine, the specificity of search results is reflected in the priority order of the returned article list depending on the nature of the search performed by the user. For instance, if a specific cDNA search is performed and there is data that matches that specific search result, the references where this match occurred are listed first before other less specific search results and demarcated in the interface for ease of reference.

#### Single-Nucleotide and Indel Variant Database Comparison

A list of 108 genetic variants provided by various clinical laboratories encountered during routine diagnostic practice were examined for content in Mastermind. Each of these variants had fewer than 5 papers identified by each lab after a search of routinely used databases and search strategies including the Human Gene Mutation Database (HGMD, Professional Edition^19^), PubMed, ClinVar^20^ (National Center for Biotechnology Information [NCBI], 2020a) and Google and/or Google Scholar^21^ (Google, 2020b). References were identified with PMID and the resulting lists were examined for concordance and sensitivity between lab-returned results and results returned after performing a Mastermind search.

#### Variant Interpretation According to ACMG Guidelines and Comparison With ClinVar

Interpretation of variants according to standard accepted ACMG published guidelines was performed and the results compared to assertions made for variants in entire genes as distributed in ClinVar. The approach to interpreting variants according to ACMG guidelines includes consultation of databases of population frequency, databases and algorithms of *in silico* models of pathogenicity prediction, and consultation of the medical literature. Details of this process are provided in the Supplementary Material. For the majority of this work, internal automation processes and curation capabilities that allow for rapid assessment of the ACMG categories were used to produce the data comprising Table 3. All the work present in this table is reproducible using the Mastermind user interface and related publicly available databases. Of note, release of many of these enhanced features into the Mastermind user interface to further enhance the utility of Mastermind in identifying and assessing the meaningfulness of each reference according to ACMG guidelines is planned for 2021.

### Copy Number Variants and Karyotypes

#### Copy Number Variant and Karyotype Identification

Using the data content in Mastermind, we have applied an exhaustive complement of regular expressions to identify all possible CNV citations from text and figures and tables. These regular expressions capture CNVs describing exon or whole gene level deletions by first identifying text describing the gene symbol or any analogous synonym (as described in the section “Methods” section above) and further identifying key words referencing deletion or amplification terms in the surrounding text. In addition, the data content of Mastermind was further evaluated to identify the mention of any cytoband (e.g., 1p36.33) as itemized in the UCSC Genome Browser Cytoband file^22^ (University of California Santa Cruz, 2020). Finally, CNVs described in a variety of karyotype nomenclatures were also identified, extracted, and disambiguated to associate them with cytobands. All user inputs or cited CNVs in the Mastermind database index – whether exon-level, gene-level, cytoband-level, or genomic coordinates in any nomenclature – were first normalized to standardized chromosomal coordinates based on data in the relevant genome build^23^ (National Center for Biotechnology Information [NCBI], 2020f) for alignment and comparison purposes. For each user input, returned matches included “Exact” matches (results from Mastermind that cited the same CNV only after normalization to genomic co-ordinates), and “Surrounding,” “Contained,” and “Intersecting” matches where Mastermind results overlapped, were contained within, or intersected with the normalized user query. For display purposes, results were prioritized according to overall size of the CNV citation, proportion of overlap with the user’s CNV query, and the match type in the following order: “Exact,” “Contained,” “Surrounding,” and “Intersecting.” The results by PMID were then output in table form with the citing PMID, the match type, and supporting information demonstrating prioritization. These results were then visualized in a custom designed stacked CNV plot. Ranges of CNVs that spanned multiple cytobands were identified and normalized to include results for all associated intervening cytobands.

#### Copy Number Variant Database Comparison

A randomly selected list of clinically relevant chromosomal cytobands was used to search Mastermind for PMIDs that contained mentions of each cytoband. The results were compared to those obtained using standard searches of PubMed. CNVD was non-functional at the time of writing, and the CNVdigest database was also not available.

### Gene Fusion Pairs

#### Gene Fusion Pair Identification

We extracted all articles mentioning any of the 507 genes included on the Illumina TruSight Fusion Gene Panel (Illumina, 2020) from Mastermind^24^. To extract the fusion pairs, we systematically searched articles that mentioned each index gene and any additional gene and prioritized articles that contained any of the three most commonly used formats for describing fusions: ‘*gene1:gene2*,’ ‘*gene1/gene2*,’ and ‘*gene1-gene2*,’ *etc.* or otherwise mentioned the two genes in the same sentence or paragraph of text. Once these fusions were extracted from individual articles, any articles mentioning the same fusion, regardless of the order of the two genes mentioned, were combined into a single entry for that specific fusion pair. The validity of each fusion pair was confirmed by manually evaluating the sentences and titles of the articles and the actual matching sentence from which the fusion pair was extracted. Identifying references describing gene fusions can be performed in the Mastermind user interface for known fusion pairs using the Boolean search capability for “gene A” and “gene B” along with categorical keywords pertaining to fusion events (“fusion,” “rearrangement,” *etc.*). Enhanced fusion gene and rearrangement search capability allowing for display of all fusion pairs for a given “gene A” search is planned for 2021. However, motivated users can currently perform searches for all fusion events involving “gene A” using the Mastermind API^25^ which offers a variety of endpoints sufficient to reproduce the results of our analyses. Additionally, a post-API script available on the Genomenon GitHub site facilitates the organization of these data^26^.

#### Gene Fusion Pair Database Comparison

To assess the ability of Mastermind to identify gene fusion events we examined results (including both fusion identification and article identification) for each of the 507 genes from the Illumina TruSight Fusion Gene Panel known to be involved in pathogenic gene fusions in cancer and compared with results obtained from the Catalog of Somatic Mutations in Cancer (COSMIC^27^) (Catalogue of Somatic Mutations in Cancer [COSMIC], 2020).

## Results

### Disease and Gene Associations and Comparison of Commercially-Available Diagnostic Panels

In total at the time of this writing, through its automated indexing process the Mastermind database includes 32,282,936 abstracts, and contains information from 7,619,062 full-text articles, and 2,599,660 supplemental files not restricted by journal type that span from 1950 to the present and are kept up to date on a weekly basis. In total, this reflects 32,799 unique journals. Each of these references was indexed for the mention of any of 41,636 named genetic entities or their associated synonyms, with 33,709 mentioned in at least one reference, 19,413 of which were found with identified genetic variants.

In order to test the validity and utility of these automatically assembled data, we sought to draw a comparison between previous gold standard, manually retrieved data by collecting the genes included on 20 distinct commercially available products. To account for variations among these panels due to size and selection criteria, we grouped and analyzed panels of similar proposed clinical use-cases, size, and type(s) of genetic lesions being detected (Supplementary Tables 2, 3). Additionally, to allow for the most equitable comparison, we compared eight hereditary cancer panels that did not include genes exclusively related to hematological cancers, ranging from 34 to 128 genes (mean panel size, 75.1 ± 33.1 genes; Supplementary Table 4A). These panels overall appeared to have the greatest degree of concordance in gene selection as well as diagnostic targets (Supplementary Tables 2, 3). Among these panels, 28 genes (13.6% of the 206 unique genes) were found on all nine panels (Supplementary Table 4A) and 98 genes (47.6%) were found on only one panel total (Supplementary Table 4B).

**FIGURE 1:**
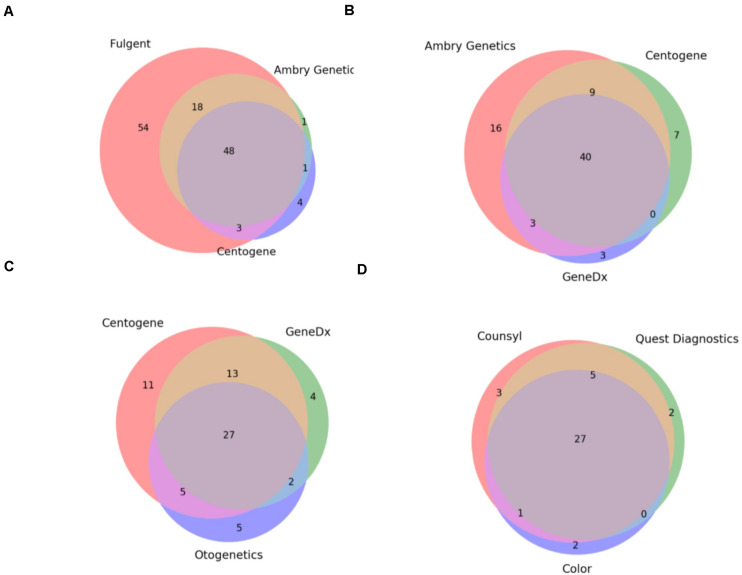
Commercially available hereditary cancer panels show significant discrepancies. **(A)** Comparison of the genes targeted by Fulgent’s Full Comprehensive Cancer Panel (123 total genes), Ambry Genetics’ CustomNext-Cancer (68 total genes), and Centogene’s CentoCancer^®^ (56 total genes). 48 genes were found to be on all 3 panels. **(B)** Comparison of the genes targeted by Ambry Genetics’ CustomNext-Cancer (68 total genes), Centogene’s CentoCancer^®^ (56 total genes), and GeneDx’s Comprehensive Common Cancer Panel (46 total genes). 40 genes were found to be on all three panels. **(C)** Comparison of the genes targeted by Centogene’s CentoCancer^®^ (56 total genes), GeneDx’s Comprehensive Common Cancer Panel (46 total genes), and Otogenetics’ Comprehensive Inherited Cancer Panel (39 total genes). 27 genes were found to be on all three panels. **(D)** Comparison of the genes targeted by Counsyl’s Reliant^TM^ Cancer Screen (expanded panel; 36 total genes), Quest diagnostics’ MYvantage^®^ Hereditary Comprehensive Cancer Panel (34 total genes), and Color Genomics’ Hereditary Cancer Test (30 total genes). 27 genes were found to be on all three panels.

Under the assumption that these 8 hereditary panels are being used for a set of identical indications (i.e., to detect germline mutations generating an increased risk for cancer development), we expected that the smallest panel would fully overlap with the next largest and so on. As such, we investigated the overlap between the panels which were of the most similar size (Figure 1). Comparison of the three largest panels – Children’s Hospital of Philadelphia’s (CHOP) Comprehensive Hereditary Cancer Gene Panel (128 genes), Fulgent’s Full Comprehensive Cancer Panel (127 genes), and Ambry Genetics’ CustomNext-Cancer (81 genes) – revealed only 63 out of 172 total unique genes (36.6%) to be concordant across all three panels (Figure 1A). The relatively low level of overlap between CHOP and Fulgent’s panel, despite there only being a difference of one gene in the size of the panel (128 and 127 genes, respectively), is especially concerning; only 89 genes were found on both panels. As seen in Figures 1A–C, however, the overlap appears to improve as the panels become smaller. Comparison of the three smallest panels – Otogenetics’ Extended Comprehensive Inherited Cancer Gene Tests (55 genes), GeneDx’s OncoGeneDx: Comprehensive Common Cancer Panel (47 genes), and Quest Diagnostics’ MYVantage Hereditary Comprehensive Cancer Panel (34 genes) – revealed 27 out of 56 unique genes (48.2%) to be concordant across all three panels (Figure 1C). Since the smallest panel has 34 genes, the observed overlap of 27 genes was 21% less than what would have been expected had these panels truly been comprehensive for the same indications. Overall, these results reflect a poor concordance rate across multiple panels used commercially for diagnosis or evaluation of risk for hereditary cancer.

Finally, these results indicate that these panels are not only inconsistent but are not truly comprehensive; often they fail to include genes with sufficient evidence for conferring cancer susceptibility or include genes that lack said evidence. A more detailed discussion of the statistical analysis of these results as well as specific gene omissions among these panels and the detailed analysis of the supporting evidence using Mastermind’s genomic association data is provided in the Supplementary Methods section.

### Single-Nucleotide and Indel Variant Identification and Comparison to ClinVar, PubMed, HGMD, and Google Scholar

The content contained in Mastermind was compared to the databases whose purpose was most analogous to that of the Mastermind database. These include ClinVar, COSMIC, and HGMD as representative variant databases for all mutations, somatic mutations in cancer, and germline mutations, respectively. Additionally, for more detailed evaluation of the indexing capabilities of Mastermind, Google Scholar and PubMed were included as representative search engines.

The overall content of Mastermind compared to manually compiled databases of genetic variants is presented in Table 1 for ClinVar, COSMIC, and HGMD. In most cases, Mastermind’s content exceeded the content of any one given manual database by 2- to 10-fold. In total, the Mastermind database includes 6,812,408 total unique variants. Fewer than 6% of these are identified in the title or abstract and the remainder are present in the full-text or supplemental files. In fact, 23% of these variants are only found in the supplemental materials. A breakdown of variants that were identified is presented by source in Supplementary Table 9A and by type in Supplementary Table 9B. This includes variants mentioned at the cDNA or protein level as well as rsIDs for both coding and non-coding variants, using HGVS standard nomenclature or not. A listing of some of the variant nomenclatures identified using Mastermind indexing is presented in Supplementary Table 10 for the most commonly cited variant, *BRAF* p.V600E. There were 99 unique ways that authors described the *BRAF* p.V600E variant, from 48,531 mentions of “BRAF V600E” to nomenclatures used only once such as “1799 T → A.” Additionally, there were 172 unique ways authors describe the CFTR p.508del (deletion) variant, from 34,091 mentions of “F508del” to single mentions of “Δ-F508” and “DeltaPhe508.” Alternate transcripts and legacy nomenclatures are also reconciled and disambiguated to ensure a maximally sensitive search result. These findings indicate that the automated approach to variant identification by Mastermind is likely to be more comprehensive than previous gold standard manual databases.

**TABLE 1:** Mastermind contains more content than HGMD, COSMIC, OncoKb, and ClinVar.

	**Mastermind**	**HGMD**		**COSMIC**		**OncoKB**		**ClinVar**	
Genes	19,413	11,320	1	238	4	671	3	32,832	6
Variants	6,812,408	298,409	8	408,000	4	5,150	3	703,806	6
Germline variants	Yes	Yes		No		Yes		Yes	
Somatic variants	Yes	No		Yes		Yes		Yes	
Journals	33,126	2,600	2	N/A		Unknown		2,977	
Full-text articles indexed	7,619,062	86,000	2	27,496	5	Unknown		70,538	7
Supplemental datasets indexed	2,599,660	N/A	2	N/A		Unknown		N/A	
Update schedule	Weekly	Quarterly		Quarterly		Quarterly		Weekly	
Free version content	Up to date	4 years old		Up to Date		<3 months old		Up to date	
Interpretation criteria	ACMG/AMP	Own criteria		AMP		Own criteria		ACMG/AMP	

*Sources ^1^http://www.hgmd.cf.ac.uk/ac/stats.php*^2^*https://www.ncbi.nlm.nih.gov/pmc/articles/PMC5429360/*^3^*https://www.oncokb.org/*^4^*https://cancer.sanger.ac.uk/cosmic/curation*^5^*https://www.ncbi.nlm.nih.gov/pmc/articles/PMC6323903/*^6^*https://www.ncbi.nlm.nih.gov/clinvar/submitters/*^7^*ftp://ftp.ncbi.nlm.nih.gov/pub/clinvar/tab_delimited/^8^https://digitalinsights.qiagen.com/wp-content/uploads/2020/10/hgmdstatistics_2020_3.pdf*

**FIGURE 2:**
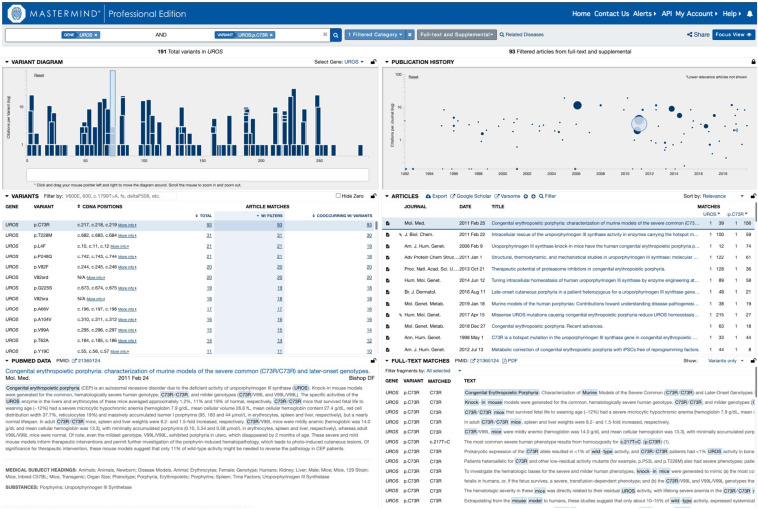
Representative screenshot of the Mastermind software interface. A representative screenshot depicting the results of a typical Mastermind variant search including the variant landscape for all variants in the searched for gene (upper left), the literature landscape results for the searched for variant (upper right; size of each icon reflects the relevance of the reference to the searched for content including categorized keywords) as well as context for the mention of the relevant search terms in the text of the specified reference (lower right).

In order to test this explicitly, we collected a list of 108 variants assembled from user submissions during software trial periods. Each of these variants were encountered in routine clinical practice and were tied directly to clinical casework. No patient specific information was provided or used for this analysis. Instead, the search results provided by each user for their routine Google Scholar, PubMed and ClinVar searches were compared to the search results for Mastermind. To assess the sensitivity and specificity of the results, each variant was searched using each of the specified resources and the references were collected and assessed for accuracy. These results are presented in full in Table 2. A representative screenshot of the Mastermind interface for a similar variant search is presented in Figure 2. For this random sampling of variants encountered in clinical practice, Mastermind identified a total of 866 references compared to 706, 467, and 194 for Google Scholar, ClinVar and PubMed, respectively. Closer inspection of these references indicated a substantial number of false positives in all but the Mastermind results. Specifically, Mastermind’s true positive percentage was 98.5% indicating that 814 references were valid. In contrast, the true positive rate for Google Scholar, ClinVar, and PubMed was 57.6, 68.8, and 45.1%, respectively, meaning the total number of true positive references for all these variants was 377, 309, and 36.

**TABLE 2:** Mastermind has increased sensitivity and specificity for evidence curation for genetic variants.

		**Mastermind**			**Google scholar**			**PubMed**			**ClinVar**						
**Gene**	**Variant**	**Total number of papers**	**Papers that are true positives (%)**	**Number of true positive references**	**Total number of papers**	**Papers that are true positives (%)**	**Number of true positive references**	**Total number of papers**	**Papers that are true positives (%)**	**Number of true positive references**	**Total number of papers**	**Papers that are true positives (%)**	**Number of true positive references**	**References only found in Master mind**	**Additional references found in Master mind**	**Reference missing in Master mind**	**Resource with reference missing in master mind**
*ABCC6*	R760W	8	100	8	10	50	5	0	–	0	0	–	0		More		
*ABCC8*	N188S	27	100	27	17	64.7	11	1	100	1	14	100	14		More		
*AGA*	A101V	6	83.3	5	13	23.1	3	0	–	0	4	100	4		More		
*ALPL*	R152H	15	93.3	14	16	62.5	10	0	–	0	12	66.7	8		More		
*AMPD1*	Q45K	78	39.7	31	0	–	0	0	–	0	0	–	0	Only	More		
*ANO10*	D615N	5	100	5	4	100	4	0	–	0	1	100	1		More		
*ANO5*	N64fs	58	100	58	1	0	0	0	–	0	0	–	0	Only	More		
*AP5Z1*	R138X	2	100	2	0	–	0	0	–	0	0	–	0	Only	More		
*APC*	A1358V	2	100	2	3	33.3	1	0	–	0	6	50	3			Missing	ClinVar
*APC*	E152fs	5	100	5	1	100	1	6	50	3	0	–	0		More		
*APC*	Q1244X	2	100	2	0	–	0	0	–	0	0	–	0	Only	More		
*APC*	R2714H	3	100	3	1	100	1	1	0	0	2	50	1		More		
*ASL*	R182X	6	100	6	5	80	4	0	–	0	2	100	2		More		
*ATM*	E3007X	1	100	1	0	–	0	0	–	0	9	0	0	Only	More		
*ATM*	Q2729H	5	100	5	2	100	2	3	0	0	3	66.7	2		More		
*ATM*	S1455R	5	100	5	3	100	3	1	0	0	3	100	3		More		
*ATP7B*	L795F	15	100	15	18	72.2	13	0	–	0	10	70	7		More		
*BMPR1A*	R254C	3	100	3	1	0	0	0	–	0	6	66.7	4			Missing	ClinVar
*BRCA1*	E638K	2	100	2	4	25	1	0	–	0	0	–	0		More		
*BRCA1*	H1283R	1	100	1	0	–	0	0	–	0	7	71.4	5			Missing	ClinVar
*BRCA1*	H239R	8	100	8	12	33.3	4	0	–	0	4	100	4		More		
*BRCA1*	L668F	22	100	22	17	70.6	12	1	0	0	14	85.7	12		More		
*BRCA1*	P897fs	12	100	12	4	100	4	0	–	0	18	22.2	4		More		
*BRCA1*	S1139I	4	100	4	2	100	2	1	0	0	5	60	3		More		
*BRCA1*	V1665M	11	100	11	13	46.2	6	7	0	0	17	70.6	12			Missing	ClinVar
*BRCA2*	Q2561R	3	100	3	1	100	1	7	0	0	4	25	1		More		
*BRCA2*	R1160K	2	100	2	1	0	0	4	0	0	2	0	0	Only	More		
*BRCA2*	R118H	6	100	6	16	18.8	3	4	0	0	5	60	3		More		
*BRCA2*	R3007G	3	100	3	4	75	3	7	0	0	1	0	0				
*BRCA2*	S683P	1	100	1	1	100	1	4	0	0	0	–	0				
*BRCA2*	Y3035S	15	100	15	18	55.6	10	4	0	0	13	76.9	10		More		
*CACN A2D1*	D1045A	3	100	3	2	100	2	31	3.2	1	0	–	0		More		
*CAPN3*	W373R	4	100	4	3	100	3	3	0	0	4	100	4				
*CCM2*	R19X	12	100	12	11	63.6	7	1	100	1	0	–	0		More		
*CDK4*	R209C	8	100	8	5	60	3	0	–	0	7	85.7	6		More		
*CHRNA4*	R495Q	1	100	1	2	50	1	0	–	0	0	–	0				
*COL4A4*	G545A	22	100	22	21	76.2	16	0	–	0	12	91.7	11		More		
*COX4I2*	R85W	2	100	2	3	66.7	2	0	–	0	1	100	1				
*DMD*	E2910V	16	100	16	12	58.3	7	1	100	1	13	69.2	9		More		
*EXT2*	A202V	2	100	2	5	40	2	0	–	0	0	–	0				
*FAH*	R174X	8	100	8	12	41.7	5	1	100	1	5	100	5		More		
*FANCA*	L1339fs	4	100	4	0	–	0	0	–	0	2	100	2		More		
*FANCC*	R179X	4	100	4	0	–	0	0	–	0	0	–	0	Only	More		
*FBN1*	C2659X	2	100	2	2	50	1	2	0	0	2	50	1		More		
*FCN3*	L117fs	27	100	27	15	80	12	0	–	0	4	100	4		More		
*FIG4*	F254fs	3	100	3	0	–	0	0	–	0	4	75	3				
*FREM2*	P187L	1	100	1	1	100	1	0	–	0	0	–	0				
*FTO*	V201I	3	100	3	2	50	1	0	–	0	0	–	0		More		
*GCDH*	G390R	4	100	4	12	33.3	4	1	100	1	4	100	4				
*HBB*	G84fs	6	100	6	0	–	0	0	–	0	16	18.8	3		More		
*HMBS*	H256Q	1	100	1	2	50	1	0	–	0	0	–	0				
*HNF1B*	V61G	13	100	13	16	50	8	2	0	0	7	85.7	6		More		
*HNRNPU*	R324G	2	100	2	2	100	2	0	–	0	13	15.4	2				
*HOGA1*	P190L	8	100	8	12	58.3	7	2	100	2	4	100	4		More		
*KCNE1*	R67H	10	100	10	17	35.3	6	1	100	1	8	62.5	5		More		
*KCNH2*	D501N	12	100	12	15	53.3	8	0	–	0	12	75	9		More		
*KCNQ1*	R397W	15	100	15	19	31.6	6	1	100	1	13	92.3	12		More		
*LDB3*	A698T	4	100	4	6	66.7	4	0	–	0	5	80	4				
*LDLR*	D304E	5	100	5	3	66.7	2	4	0	0	13	7.7	1		More		
*LDLR*	D482G	1	100	1	4	25	1	7	0	0	1	100	1				
*LDLR*	D492H	3	66.7	2	1	0	0	0	–	0	3	0	0	Only	More		
*LDLR*	D622G	1	100	1	1	100	1	7	0	0	2	50	1				
*LDLR*	F73C	1	100	1	1	100	1	7	0	0	0	–	0				
*LIPT1*	S292X	5	100	5	1	100	1	1	100	1	3	33.3	1		More		
*LRRK2*	R521G	3	100	3	20	10	2	0	–	0	1	0	0		More		
*MCCC1*	M325R	6	100	6	3	66.7	2	0	–	0	5	60	3		More		
*MLH1*	K461N	3	100	3	2	100	2	0	–	0	0	–	0		More		
*MLH1*	S577L	3	100	3	1	0	0	0	–	0	5	80	4			Missing	ClinVar
*MLH1*	V664del	2	100	2	0	–	0	0	–	0	0	–	0	Only	More		
*MMACHC*	Y130C	6	100	6	4	100	4	1	100	1	2	100	2		More		
*MMP21*	W401X	1	100	1	0	–	0	0	–	0	1	100	1				
*MSH2*	A636V	1	100	1	2	50	1	0	–	0	5	0	0				
*MSH2*	Q377X	2	100	2	9	0	0	0	–	0	0	–	0	Only	More		
*MSH2*	R171K	1	100	1	6	16.7	1	2	0	0	2	100	2			Missing	ClinVar
*MSH6*	S564X	1	100	1	3	0	0	0	–	0	4	25	1				
*MSH6*	T767I	4	100	4	9	33.3	3	1	100	1	3	33.3	1		More		
*MUTYH*	W174X	3	100	3	6	16.7	1	0	–	0	0	–	0		More		
*MYBPC3*	S858N	10	100	10	10	50	5	1	0	0	9	88.9	8		More		
*MYH7*	D778E	15	100	15	16	62.5	10	1	0	0	9	88.9	8		More		
*MYH7*	I530V	2	100	2	4	25	1	5	0	0	3	100	3			Missing	ClinVar
*MYO7A*	R336C	1	100	1	0	–	0	0	–	0	0	–	0	Only	More		
*MYO7A*	T165M	8	100	8	15	60	9	2	100	2	9	100	9			Missing	Google, ClinVar
*MYPN*	P1112L	18	100	18	9	88.9	8	1	100	1	8	87.5	7		More		
*MYPN*	Y20C	15	100	15	24	41.7	10	1	100	1	6	100	6		More		
*OCA2*	A334T	1	100	1	0	–	0	0	–	0	12	0	0	Only	More		
*OCA2*	N489D	21	100	21	24	79.2	19	1	100	1	6	83.3	5		More		
*PAH*	F39del	58	100	58	8	100	8	0	–	0	20	90	18		More		
*PALB2*	T226A	1	100	1	0	–	0	0	–	0	0	–	0	Only	More		
*PMS2*	E473K	1	100	1	7	14.3	1	0	–	0	3	33.3	1		More		
*PMS2*	E661K	1	100	1	0	–	0	1	0	0	3	66.7	2			Missing	ClinVar
*PMS2*	L729fs	15	100	15	0	–	0	0	–	0	0	–	0	Only	More		
*PRODH*	A455S	6	100	6	7	71.4	5	1	100	1	2	100	2		More		
*PTCH1*	G38A	1	100	1	6	0	0	0	–	0	0	–	0	Only	More		
*PTEN*	C296X	3	100	3	2	100	2	0	–	0	0	–	0		More		
*RAD50*	R138X	4	100	4	6	33.3	2	0	–	0	3	66.7	2		More		
*SETX*	G2169G	1	100	1	1	100	1	36	0	0	0	–	0				
*SHOX*	R147H	5	100	5	8	62.5	5	1	100	1	0	–	0				
*SLURP1*	W15R	19	100	19	22	54.5	12	4	100	4	5	100	5		More		
*SMPD1*	L180fs	1	100	1	0	–	0	0	–	0	4	25	1				
*SOS1*	P340S	2	100	2	4	50	2	1	100	1	3	66.7	2				
*SURF1*	D202H	15	100	15	5	40	2	0	–	0	0	–	0		More		
*TMC1*	R389X	17	100	17	14	78.6	11	1	100	1	3	100	3		More		
*TMPRSS3*	A306T	18	100	18	23	65.2	15	6	100	6	7	100	7		More		
*TP53*	E343Q	2	100	2	1	100	1	3	0	0	0	–	0		More		
*TSC2*	A84V	5	80	4	5	20	1	0	–	0	1	100	1		More		
*TTR*	E109K	6	100	6	9	55.6	5	0	–	0	0	–	0		More		
*TTR*	E74Q	5	80	4	8	25	2	0	–	0	3	100	3		More		
*WNK4*	P556T	4	100	4	7	57.1	4	1	100	1	0	–	0				
Total/average		866	98.5	814	706	57.6	377	194	45.1	36	467	68.8	309	15	76	9	
Sensitivity per variant				100.0%			76.9%			22.2%			63.9%				
Specificity per article			98.5%			57.6%			45.1%			68.8%					

When the data was examined on a per case basis, there were 76 variants for which additional references were returned beyond the number contained in the other resources, reflecting a benefit of using Mastermind in 70% of cases. Moreover, there were 15 variants where Mastermind was the only resource that contained references. This indicates that 13.9% of these cases showed empirical evidence only in Mastermind which would have otherwise been missed if using Google Scholar, ClinVar, and PubMed alone. On the other hand, there were 13 references that were identified in these other resources that were not identified in Mastermind representing a low false negative rate of 1.6% (or 13 out of 827 references) compared to a relatively high false positive rate of 95.6% for PubMed, 62.6% for ClinVar, and 54.4% for Google Scholar. In 8 out of 9 of these cases where Mastermind was missing a reference, there was only 1 additional reference found in ClinVar. Google Scholar had an additional reference in only a single case compared to Mastermind. Integration of the publicly available ClinVar dataset is a planned future feature of Mastermind at which time no references in ClinVar will not be included in Mastermind. Overall, these results demonstrate the superiority of the Mastermind database for both sensitivity and specificity of returned reference matches.

To examine in more depth the value of the references returned for any of these variants, we sought to curate complete sets of variants for a randomly selected cohort of genes associated with constitutional disease (according to the ACMG guidelines) as identified using Mastermind. We chose 27 genes across a variety of diseases and curated the assembled evidence across all variants for each of the genes. These interpretation results were compared with results per variant in ClinVar. In total, Mastermind identified 16,287 total variants whereas ClinVar identified 7,245, reflecting a 2.2-fold increase in variants when using Mastermind (average 9.7 across all 27 genes, reflecting a wide range from 0.9 to 127-fold increase). When the nature of the individual variants was examined using the ACMG guidelines for assessing pathogenicity, Mastermind identified 4,687 Pathogenic or Likely Pathogenic variants, each with supporting evidence from the empirical literature (ranging from one supplemental reference to more than 8,000 articles). In contrast, ClinVar identified only 2,262 Pathogenic or Likely Pathogenic variants, representing a 2.1-fold increase in disease-causing variants when using Mastermind. Closer scrutiny of this data also suggested that many of the pathogenic variants identified in ClinVar lacked reference citations, calling into question the validity of these user submitted interpretations (data not shown). These results are presented in full in Table 3. Overall, these results indicate that Mastermind can identify, on average, 9.7-fold more variants than the previous gold standard ClinVar, including 4.8-fold more pathogenic variants. This is in addition to allowing for a more complete understanding of the clinical and functional evidence supporting the pathogenicity and actionability of each variant. Mastermind is therefore a superior resource for identifying evidence with which to interpret genetic variants according to industry-accepted standards including ACMG guidelines.

**TABLE 3:** Mastermind contains more disease-causing variants than ClinVar.

		**Mastermind**							**Total**	**Disease-causing variants**	**ClinVar**							
					
	**Gene**	**Total**	**Pathogenic**	**Likely pathogenic**	**Conflict**	**Variant of uncertain significance**	**Likely benign**	**Benign**	**MM/CV**	**MM/CV**	**Total**	**Pathogenic**	**Likely pathogenic**	**Conflict**	**Variant of uncertain significance**	**Likely benign**	**Benign**	**Other**
(1)	*TP63*	753	30	93	0	513	0	117	2.4	0.9	318	103	33	6	74	64	38	0
(2)	*PHEX*	478	239	74	0	140	3	22	1.2	1.0	406	251	66	5	36	26	20	2
(3)	*BBS9*	585	23	51	0	376	0	135	1.9	1.1	316	60	6	31	104	53	62	0
(4)	*ALMS1*	1104	338	75	0	634	1	56	0.6	1.1	1918	145	223	82	875	459	132	2
(5)	*IFT172*	254	9	19	0	209	0	17	1.9	1.2	134	19	4	2	24	52	33	0
(6)	*ATP7B*	1310	366	282	14	566	30	52	1.1	1.3	1230	245	240	97	353	190	97	8
(7)	*NTRK2*	302	1	6	0	73	5	217	9.2	1.4	33	5	0	1	4	18	4	1
(8)	*THRB*	525	30	69	0	297	0	129	2.3	1.4	232	59	10	6	82	52	23	0
(9)	*EDA*	470	105	126	0	221	0	18	2.1	1.5	226	101	50	6	44	11	13	1
(10)	*BBS2*	384	36	69	0	259	0	20	2.1	1.7	187	35	28	14	62	36	12	0
(11)	*GBA*	852	104	182	2	551	3	10	3.6	1.9	236	114	35	10	45	9	11	6
(12)	*CEP290*	766	3	349	0	375	0	39	1.3	2.2	592	113	50	54	165	127	65	14
(13)	*NECTIN1*	320	1	12	0	247	4	56	6.5	2.2	49	6	0	0	0	17	26	0
(14)	*IKBKG*	703	81	116	1	501	1	3	7.6	2.8	93	56	14	3	7	5	1	7
(15)	*MKKS*	283	46	42	4	161	4	26	2.6	2.8	108	25	6	3	44	15	14	1
(16)	*TARDBP*	303	15	88	2	187	1	10	3.5	5.2	86	18	2	4	42	10	10	0
(17)	*WDPCP*	974	0	36	0	887	49	2	9.5	6.0	103	4	2	7	52	23	13	2
(18)	*POMC*	287	48	34	0	183	2	20	6.2	6.3	46	12	1	5	19	6	3	0
(19)	*CFI*	349	45	70	2	189	1	42	3.6	6.8	97	10	7	1	27	22	21	9
(20)	*BDNF*	632	0	17	0	549	0	66	27.5	8.5	23	0	2	0	3	8	10	0
(21)	*CFH*	845	102	127	10	416	4	186	6.6	9.5	129	22	2	1	36	33	26	9
(22)	*C3*	313	25	61	4	141	2	80	2.2	9.6	140	3	6	7	37	47	35	5
(23)	*LEPR*	692	42	47	0	261	4	338	11.0	9.9	63	6	3	2	33	4	15	0
(24)	*PCSK1*	414	31	84	2	235	7	55	4.7	12.8	89	7	2	3	51	22	4	0
(25)	*LRRK2*	962	29	126	37	460	13	297	3.1	12.9	308	10	2	10	178	61	47	0
(26)	*MC4R*	919	136	408	33	304	32	6	11.6	13.9	79	33	6	4	20	8	3	2
(27)	*MC3R*	508	21	118	10	346	4	9	127.0	N/A	4	0	0	0	0	2	0	2
	Total	16287	1906	2781	121	9281	170	2028	9.7	4.8	7245	1462	800	364	2417	1380	738	71

### Copy Number Variant and Karyotype Abnormality Identification and Comparison to PubMed

A major challenge when interpreting the pathogenicity of CNVs is the difficulty associated with finding empirical and clinical evidence from the medical literature necessary for making determinations about the diagnostic, prognostic and/or therapeutic actionability of a patient’s CNV profile. We took advantage of Mastermind’s ability to identify associations from the empirical literature by searching for CNV data whether described at the karyotypic level (as from cytogenetic and chromosomal banding techniques), the cytoband level (as from a microarray result), the whole gene or gene exon level (such as when describing more focused or specific deletions or amplifications and their effects), or lastly, at the genomic co-ordinate level.

To assess the benefit of this new approach to evidence assembly for CNVs, we chose 10 randomly selected, clinically relevant cytobands from among the total 863 named cytobands in the human genome and compared search results in Mastermind to results from PubMed. The results of this comparison are provided in Table 4. The unique number of references identified in Mastermind ranged from 52 to 4,808 (average 1,018) for exact matches compared to 28 to 3,322 (average 881) for exact matches in PubMed. This reflects an average 4.0-fold increase in references identified using Mastermind and resulted from a deeper reach into the full-text compared to PubMed. Additionally, Mastermind’s superior ability to recognize appropriate content without the requirement that there be an exact match for a given query resulted in vastly more references compared to PubMed. For instance, deletions or amplifications described solely at the gene level are also critical to understanding the significance of clinical and functional evidence needed to properly interpret a CNV and would not be available in a PubMed CNV search. The increase in references identified in Mastermind when these additional matches were considered reflected a 43.9-fold increase compared to PubMed results containing only exact matches – between 1,099 to 13,363 prioritized references (average 4,495) for total matches in Mastermind. In fact, only 1 in 8 of the search results in Mastermind were from entries that reflected a exact match of the searched for CNV (12.5% “Exact”; 3.3–25.8%) indicating that the majority of relevant results were previously unobtainable in PubMed or other search strategies such as those using Google Scholar that require an exact match. For the representative CNVs depicted in Table 4, the majority of the references comprising the search results described the CNV as either a cytoband or karyotype (79.4%; 69.8–89.4%) with the balance of the references referring to specific gene exon deletions/amplifications (2.2%; 0.7–4.4%) or entire gene deletion/amplifications (18.4%; 9.9–27.2%). The breakdown of the match types based on article count is depicted in Supplementary Tables 11A,B.

**TABLE 4:** Mastermind has increased sensitivity for identifying references for literature curation for CNVs.

**Cytoband**	**Chr**	**Start**	**End**	**Disease name(s)**	**Total references identified in Mastermind**	**Total exact matches identified in Mastermind**	**Total references identified in PubMed**	**Ratio of references identified in Mastermind/PubMed**	**Ratio of references containing exact matches in Mastermind/PubMed**	**Key references**
11q23	chr11	110,600,000	121,300,000	Jacobsen syndrome, 11q23 deletion syndrome	7,926	1,625	490	16.2	3.3	31895838, 29307309
14q32.2	chr14	95,800,000	100,900,000	Kagami–Ogata syndrome	2,470	147	30	82.3	4.9	30232357
15q11.2	chr15	20,500,000	25,500,000	15q11.2 BP1–BP2 microdeletion syndrome, Burnside-Butler syndrome	4,095	850	268	15.3	3.2	30342661, 28254235, 25689425, 31207912
17p11.2	chr17	16,100,000	22,700,000	Smith–Magenis syndrome	3,604	1,093	403	8.9	2.7	29138588, 20301487
17q12	chr17	33,500,000	39,800,000	17q12 microdeletion syndrome, 17q12 deletion and duplication syndrome, 17q12 deletion syndrome	4,677	619	169	27.7	3.7	27409573, 29060963, 30032214, 32219821
18q21.1	chr18	45,900,000	50,700,000	Pitt–Hopkins syndrome	2,525	344	71	35.6	4.8	28520343, 22934316
22q11.2	chr22	17,400,000	25,500,000	22q11.2 Deletion syndrome (22q11.2DS)	13,363	4,808	2,086	6.4	2.3	32117416, 20301696, 30380194
22q13.3	chr22	43,800,000	50,818,467	Phelan-McDermid syndrome, 22q13.3 deletion syndrome	2,882	545	116	24.8	4.7	20301377, 26350728
2q22.3	chr2	143,400,000	147,900,000	Mowat–Wilson syndrome	1,099	52	11	99.9	4.7	19215041, 20301585
4q35.1	chr4	182,300,000	186,200,000	Terminal chromosome 4q deletion syndrome	2,312	100	19	121.7	5.3	24962056
				Average	4,495	1,018	366	43.9	4.0	
				Min	1,099	52	11	6.4	2.3	
				Max	13,363	4,808	2,086	121.7	5.3	

Identifying these mentions using the Mastermind index and associating the results with additional ontological entities or key terms will provide a powerful tool to expedite the interpretation of CNV data at the same time it will likely improve diagnostic yield.

### Fusion Gene Identification and Comparison to COSMIC

**FIGURE 3:**
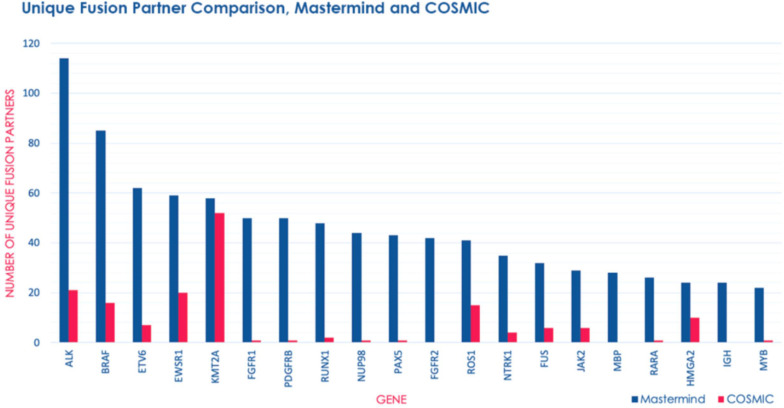
Mastermind identifies a significant number of additional fusion gene partners than COSMIC. The number of unique fusion partners identified by Mastermind (blue) compared to results for those same genes identified in COSMIC (pink) from among genes routinely commercially tested for fusion events using the Illumina TruSight RNA fusion Sequencing Panel.

In order to determine whether Mastermind could serve as a more comprehensive source for documented fusions, we developed a process to automatically retrieve fusion genes from our database of approximately 6.5 million full-text genomic articles. To focus our study on fusion events of clinical significance, we restricted our analysis to the 507 genes comprising the Illumina TruSight Fusion Gene Panel (Illumina, 2020). The result of this analysis for the representative gene *ALK* is presented in Supplementary Table 12. For these 507 genes, we discovered a total of 1,896 unique fusion pairs cited in the scientific literature, all of which were manually validated. This represents a 538% increase in yield over the 297 unique fusions in the COSMIC database (for detailed results for top genes see Figure 3) (Catalogue of Somatic Mutations in Cancer [COSMIC], 2020). Additionally, even for those fusions also identified in COSMIC, Mastermind typically found significantly more references per fusion gene pair (Table 5).

**TABLE 5:** Mastermind has increased sensitivity for identifying references for literature curation for gene fusion events.

**Fusion event**	**Number of references identified in Mastermind**	**Number of references identified in COSMIC**	**Ratio of references identified in Mastermind/COSMIC**
*RUNX1-RUNX1T1*	2706	55	49.2
*ETV6-JAK2*	365	8	45.6
*ETV6-RUNX1*	1940	43	45.1
*PML-RARA*	1477	42	35.2
*HEY1-NCOA2*	51	2	25.5
*TPM3-ROS1*	22	1	22.0
*SLC45A3-ELK4*	37	2	18.5
*FGFR3-TACC3*	238	14	17.0
*TMPRSS2-ERG*	1895	114	16.6
*ETV6-PDGFRB*	252	16	15.8
*NPM1-ALK*	1322	86	15.4
*TCF3-PBX1*	796	53	15.0
*EPC1-PHF1*	43	3	14.3
*FUS-ERG*	228	16	14.3
*NAB2-STAT6*	168	12	14.0
*EWSR1-PBX1*	28	2	14.0
*PAX3-NCOA2*	28	2	14.0
*PAX3-NCOA1*	41	3	13.7
*FUS-CREB3L1*	66	5	13.2
*ETV6-NTRK3*	641	49	13.1
*EWSR1-CREB1*	143	11	13.0
*MN1-ETV6*	87	7	12.4
*CLTC-TFE3*	48	4	12.0
*PAX3-FOXO1*	835	72	11.6
*ETV6-ABL1*	283	25	11.3
*MEAF6-PHF1*	33	3	11.0
*DNAJB1-PRKACA*	66	6	11.0
*IRF2BP2-CDX1*	11	1	11.0
*SS18L1-SSX1*	11	1	11.0
*PRCC-TFE3*	119	11	10.8
*FUS-FEV*	32	3	10.7
*NUP214-ABL1*	167	16	10.4
*CCDC6-RET*	1652	164	10.1
*SLC45A3-ERG*	30	3	10.0
*STRN-ALK*	49	5	9.8
*CIC-FOXO4*	29	3	9.7
*FUS-DDIT3*	461	48	9.6
*ASPSCR1-TFE3*	314	33	9.5
*JAZF1-PHF1*	57	6	9.5
*EWSR1-NFATC2*	19	2	9.5
*EWSR1-PATZ1*	19	2	9.5
*FUS-CREB3L2*	112	12	9.3
*SQSTM1-ALK*	28	3	9.3
*FGFR3-BAIAP2L1*	27	3	9.0
*PAX7-FOXO1*	496	56	8.9
*FUS-ATF1*	42	5	8.4
*SLC45A3-BRAF*	25	3	8.3
*EWSR1-FLI1*	1387	167	8.3
*NONO-TFE3*	33	4	8.3
*EWSR1-ATF1*	384	47	8.2
*EWSR1-POU5F1*	49	6	8.2
*RANBP2-ALK*	89	11	8.1
*EWSR1-ZNF384*	8	1	8.0
*PCM1-JAK2*	135	17	7.9
*EWSR1-ETV1*	93	12	7.8
*PAX5-JAK2*	30	4	7.5
*TPM4-ALK*	99	14	7.1
*BRD3-NUTM1*	42	6	7.0
*JAZF1-SUZ12*	131	19	6.9
*TPM3-ALK*	185	27	6.9
*SEC31A-ALK*	33	5	6.6
*SS18-SSX2*	904	137	6.6
*MSN-ALK*	39	6	6.5
*EWSR1-DDIT3*	173	27	6.4
*CBFA2T3-GLIS2*	50	8	6.3
*EWSR1-ERG*	559	91	6.1
*EWSR1-WT1*	325	55	5.9
*CRTC1-MAML2*	212	36	5.9
*HIP1-ALK*	16	3	5.3
*TFG-ALK*	130	25	5.2
*EML4-ALK*	2566	518	5.0
*SET-NUP214*	74	15	4.9
*CLTC-ALK*	122	25	4.9
*ATIC-ALK*	97	20	4.9
*EWSR1-ETV4*	63	13	4.8
*PPFIBP1-ALK*	14	3	4.7
*CARS-ALK*	28	6	4.7
*EWSR1-FEV*	79	17	4.6
*HAS2-PLAG1*	18	4	4.5
*EWSR1-SP3*	9	2	4.5
*KIF5B-RET*	230	53	4.3
*SS18-SSX4*	82	19	4.3
*KIF5B-ALK*	133	31	4.3
*ERC1-RET*	38	9	4.2
*COL1A1-PDGFB*	217	52	4.2
*HMGA2-RAD51B*	20	5	4.0
*TCF12-NR4A3*	16	4	4.0
*COL1A2-PLAG1*	12	3	4.0
*HMGA2-LPP*	63	16	3.9
*SS18-SSX1*	501	136	3.7
*PCM1-RET*	25	7	3.6
*SSBP2-JAK2*	14	4	3.5
*TAF15-NR4A3*	37	11	3.4
*EWSR1-NR4A3*	71	22	3.2
*SLC45A3-ETV1*	9	3	3.0
*TMPRSS2-ETV1*	100	34	2.9
*EZR-ROS1*	53	19	2.8
*SFPQ-TFE3*	20	8	2.5
*TMPRSS2-ETV4*	47	19	2.5
*PAX8-PPARG*	183	77	2.4
*TRIM24-RET*	22	10	2.2
*SLC34A2-ROS1*	91	44	2.1
*EWSR1-SMARCA5*	4	2	2.0
*EWSR1-NFATC1*	2	1	2.0
*EWSR1-YY1*	2	1	2.0
*FGFR1-ZNF703*	2	1	2.0
*HMGA2-CCNB1IP1*	2	1	2.0
*KMT2A-MLLT3*	58	36	1.6
*KMT2A-MLLT1*	35	33	1.1
*KMT2A-FOXO4*	2	2	1.0
*PPFIBP1-ROS1*	1	1	1.0
*KMT2A-SEPT5*	1	1	1.0
*HMGA2-COX6C*	2	2	1.0
*NUP107-LGR5*	1	1	1.0
*ERC1-ROS1*	1	1	1.0
*ETV6-ITPR2*	1	1	1.0
*KMT2A-AFF1*	63	74	0.9
*HMGA2-LHFP*	2	3	0.7
*KMT2A-MLLT10*	15	26	0.6
*KMT2A-MLLT6*	5	10	0.5
*KMT2A-ELL*	8	17	0.5
*KMT2A-TET1*	2	5	0.4
*KMT2A-MLLT11*	2	7	0.3
*KMT2A-SEPT6*	3	13	0.2
*KMT2A-CREBBP*	1	8	0.1
*PRKAR1A-RET*	4	38	0.1
*KMT2A-EPS15*	1	14	0.1

Additionally, we were able to identify fusions that are not found in the two most commonly used databases for documented fusion events: COSMIC and TCGA. *NPM1-TYK2* is an example for which there is a known pathogenic disease association and a potential therapy. This fusion was discovered in 2014 and found to be recurrent in cutaneous CD30-positive lymphoproliferative disorders (Velusamy et al., 2014). Because the fusion event involves activation of the JAK-STAT pathway member TYK2, it represents a potentially targetable fusion event.

The top 5 most common fusion partners we discovered were *ALK* (*n* = 94 unique fusion partners), *BRAF* (*n* = 74 unique fusion partners), *ETV6* (*n* = 62 unique fusion partners), *EWSR1* (*n* = 53 unique fusion partners), and *RUNX1* (*n* = 50 unique fusion partners). Overall, the top 20 most common fusion partners represented 28.9% of the total fusion partners identified (Supplementary Table 13). Based on the publication date of the articles describing these fusions, we also discovered that both the total number of articles describing fusions and the number of articles describing novel fusions involving these genes has experienced a relatively constant increase from 1987 to 2018 (Supplementary Table 14). This trend represents a steady increase in the recognition of fusion genes in clinical diagnostic labs and research laboratories.

In 2018 alone, over 200 novel gene fusions were found across nearly 500,000 newly published scientific articles containing genomic content. In 2019, we identified 31 novel fusions across 21 articles involved in the genesis of multiple cancer types (Supplementary Table 15). Several of these fusions were unique, having been discovered in an individual patient. *BRAF-SEPT3* is a novel fusion discovered in one patient with melanoma and conferred the least proliferative but most invasive phenotype of the three BRAF fusions that were evaluated, as well as a low treatment response to MAPK inhibitors (Turner et al., 2019). Similarly, *NTRK3-KHDRBS1* was discovered in an infant with a CD34-positive spindle-cell skin tumor and is of particular interest due to the more recent identification of NTRK3 fusions as drivers in the development of rare cancer types, as well as the recent development of TRK inhibitors (Tallegas et al., 2019). Other fusions were detected in more than one case. *LPP-RFC4*, is a recurrent but non-pathogenic fusion in mucinous breast cancer (Pareja et al., 2019) and *CBFA2T3-PAX5* is a recurrent fusion in a high-risk subtype of B-progenitor acute lymphoblastic leukemia (Gu et al., 2019). Overall, these findings illustrate an increased interest in the role of fusions in cancer generally, as well as emphasize the true breadth and heterogeneity of the fusion landscape.

## Discussion

### Benefits of Mastermind for Identifying Genetic Associations for Designing Gene Panels

Our results indicate that there is substantial discordance between commercially available comprehensive hereditary cancer panels that claim to be useful for the same purpose – to detect germline mutations associated with an increased risk for cancer development. These panels included genes that were not adequately supported as cancer predisposition or cancer-causing genes as well as failed to include genes that *are* adequately supported as cancer predisposition genes. These results underscore the need to carefully examine the biomarker content of any commercial gene panel to ensure there is adequate diagnostic utility, especially pertaining to panels marketed as comprehensive.

As these panels are being utilized within clinical settings as opposed to laboratory research, the inclusion of genes with contradictory or inconclusive evidence is also concerning. In order for the panels to be clinically useful, they must produce actionable information; a gene of undetermined significance in relation to cancer susceptibility cannot produce this information. Moreover, the absence of a clinically meaningful gene from a sequencing panel will result in clinically consequential false negatives.

These discrepancies in comprehensive hereditary cancer panels could potentially be attributed to two factors – one, a lack of consensus on what a ‘comprehensive’ cancer panel should consist of (e.g., common cancer types versus more rare cancer predisposition syndromes) and two, a gap in knowledge where recently described and/or lesser known cancer predisposition genes simply are missed during the labor-intensive, manual curation process. False positives – namely, mistakenly selected genes with little to no evidence supporting a disease-association – could also be attributed to lack of a sufficiently comprehensive knowledgebase to eliminate remaining suspicions about a gene’s potential role in cancer development and/or the exact nature of that role.

Thus, we propose the use of more systematic and comprehensive approaches to evidence-based gene biomarker selection to come closer to a consensus in the selection of biomarkers. Our results have indicated that the use of Mastermind to systematically organize and evaluate evidence from the genetic literature was a useful mechanism to assess the validity of any given biomarker candidate and may help facilitate more automated approaches to evidence-based gene panel design. Finally, systematic examination of the evidence supporting the inclusion of biomarker candidates provided a useful mechanism to assess the validity of any given biomarker candidate and may help facilitate more automated approaches to evidence-based gene panel design.

### Benefits of Mastermind for Clinical Interpretation of Single-Nucleotide and Indel Variants

With the increasingly widespread adoption of rapid and inexpensive genome sequencing assays in both the clinic and in research, it is becoming clearer that the manual approach to curating and interpreting this information is not scalable. Particularly in diagnostic labs where the reproducibility of the interpretations is paramount, the dramatic increase in information that needs to be accessible in the empirical evidence is a major bottleneck. Moreover, as the trend toward larger and larger panels culminates in full exome or genome sequencing of patients, the need to have a reliable, comprehensive and automatic understanding of this published information is critical.

Attempts to aid variant interpretation beyond using PubMed or Google Scholar have been made, including by ClinVar, a freely available public archive of variants and their interpretations (Landrum et al., 2018). ClinVar relies on user submissions to curate interpretations for variants and provide supporting evidence from the empirical literature. However, due to inconsistency among clinical laboratories even with the use of ACMG criteria and applying similar search strategies to identify and interpret the empirical evidence, the process remains error-prone (Gradishar et al., 2017; Harrison et al., 2017). The opportunity for open communication between clinical laboratories that ClinVar provides, however, has been shown to increase concordance (Harrison et al., 2017). Another tool built as an aid for variant interpretation, specifically for applying ACMG criteria, is InterVar (Li and Wang, 2017). However, InterVar only provides support for 18 of the 28 ACMG criteria and relies solely on existing public databases, including ClinVar, which have been shown to suffer from inadequacies and inconsistencies (Harrison et al., 2017; Yen et al., 2017). Moreover, it does not provide access to critical information from the genetic literature that can provide support for the remaining 10 criteria as well as assist in resolving the inconsistencies which exist in these databases. In order to improve on this, the information contained within the genetic literature needs to be captured and presented in an organized, systematic manner.

In this work, we have shown how Mastermind’s automated approach to data aggregation, organization and annotation solves these problems by examining results for individual variant curation using Mastermind and comparing its performance to previously used resources like PubMed, Google Scholar, and ClinVar. Mastermind outperformed each of these resources both in terms of sensitivity as well as specificity. In fact, in nearly 14% of these cases, Mastermind was the only resource that contained information necessary to adequately interpret the clinical significance of the variant. This suggests that use of Mastermind on previously unresolved cases is likely to significantly improve diagnostic yield by allowing VUS results to be converted to more clinically meaningful results. Anecdotal evidence from our users indicates that this is indeed true (manuscripts in preparation).

Moreover, when the variant content for entire genes was examined using the data contained in Mastermind, the data indicated a dramatic increase in the number of variants that were determined to be pathogenic as compared to ClinVar indicating a failure of the manual approach even when facilitated by crowd-sourced curation. The limitations of the model employed by ClinVar are apparent when considering the lack of evidence and reference citations supporting any of the user-submitted results resulting in frequently discordant results. Moreover, the challenge of keeping up to date with newly published information for each previously entered variant in addition to newly published variants will be a continual rate-limiting step in maintaining this resource to ensure its accuracy and utility. We propose Mastermind as an alternative to ClinVar and similar manually curated databases including HGMD for coalescing this information as Mastermind has a much more comprehensive collection of informative variants and the needful empirical evidence for each.

Finally, the ability to more thoroughly automate the curation and interpretation of genetic variants is achievable by leveraging Mastermind’s superior and more comprehensive data and puts the possibility of rapidly curating the entire genome of all hypothetical variants within reach – work which is forthcoming.

### Evidence for Clinical Assessment of CNVs and Gene Fusions

While curating single nucleotide and smaller insertion deletion variants solves much of the challenge associated with interpreting genetic sequencing assays, a significant proportion of cases have disease-causing and diagnostically useful structural alterations whose contribution to clinical care must be taken into account. These larger structural alterations – CNVs and fusion events – contribute to clinical decision-making and treatment decisions in 10–25% of clinical cases for either constitutional disease or somatic cancers and therefore are an important consideration for a large number of otherwise unresolved cases.

Identifying evidence for CNVs is challenged by a lack of appropriate databases and searching independently for this evidence is especially difficult given the myriad of different nomenclature types used by authors of empirical studies to describe these changes. Not only does this searching take a great deal of time for clinical scientists, geneticists and pathologists, but the subjectivity of the search and the restriction of exact match requirements using other search techniques makes this process prone to numerous false negatives and irreproducibility across different labs or even different searchers within the same lab. We therefore applied the Mastermind indexing infrastructure to identify and categorize CNVs. Our approach included looking for CNVs mentioned by authors as deletions or amplifications according to specific genomic coordinate ranges, affecting specific genes or gene exons, involving chromosomal cytobands, or otherwise embedded in the descriptions of karyotypic results. When results for representative CNV searches in Mastermind were compared with those in PubMed, Mastermind identified several-fold more results, indicating an improved sensitivity of search results. Moreover, Mastermind’s ability to cross-index any of these results with additional annotations including diseases, phenotypes, therapies and associated clinical and functional keywords reflects a uniquely powerful capability to improve the specificity of the search results as well.

Whereas there is no suitable database of clinically relevant CNVs for an adequate comparison, there *are* manually assembled databases of clinically relevant fusion gene events. We compared the data content in these databases with the content in Mastermind for fusion events and the results have indicated that COSMIC the most widely utilized database for fusion events in cancer– are insufficient sources for comprehensive documentation of fusion events. COSMIC lacks the literature support needed to ensure that the database is fully inclusive of all documented fusions, as evidenced by fusions Mastermind identified that were present in neither database. Additionally, the fusions in COSMIC are aggregated from large sequencing studies which prevents the more detailed curation that is possible through analysis of the literature. Through the use of Mastermind, we were able to discover several-fold more fusions than were documented in the COSMIC database, all of which were manually validated to be clinically relevant.

Since the number of fusions being documented is steadily increasing, the need for literature support in documenting these fusions will similarly increase. The literature can ultimately provide a more detailed understanding of the effects of fusion events than is currently possible using sequencing-based datasets such as COSMIC. Without the literature, the mechanistic evidence behind a fusion’s role in cancer development cannot be fully elucidated, preventing the development and use of effective targeted therapies. Moreover, these fusions being aggregated from large patient sequencing studies, without more detailed curation, introduces the potential for inclusion of incidental fusion events that do not drive disease, non-functional read-through fusions, or sequencing and bioinformatic artifacts.

Overall, our results have indicated that Mastermind, with its ability to systematically organize and analyze the medical/genetic literature, can serve as a more comprehensive source for the documentation of structural alterations including CNVs and fusion events in both constitutional diseases and somatic cancer.

### Overall Conclusion

Altogether, these findings demonstrate that Mastermind’s novel automated approach to extraction, organization, and annotation of genomic association data from the primary scientific literature is of superior sensitivity compared to manual database assembly methods without compromising specificity. Moreover, we demonstrate Mastermind’s superior utility for designing and assessing gene panels as well as identifying references for and arriving at interpretations of genetic variants including structural alterations such as CNVs and fusion events. New information about disease-gene associations and an increasing number of additional insights about previously identified genetic variants and newly discovered variants are being published on a weekly basis. As such, the reliable and scalable solution provided by Mastermind is essential to ensure patients receive adequate care based on their disease’s genomic profile.

## Data Availability Statement

The datasets generated for this study can be found in online repositories. The names of the repository/repositories and accession number(s) can be found in the article/ Supplementary Material.

## Author Contributions

LC, RT, KE-J, ML, and MK: experimental design and methodology. GC, LC, DN, RS and RT: data collection and organization. LC and RT: formal data analysis. MK and SS: software design. LC and MK: manuscript preparation and review. All authors contributed to the article and approved the submitted version.

## Conflict of Interest

GC, LC, KE-J, DN, ML, MK, RS, and SS are founders and/or employees and own stock in Genomenon, Inc. which is commercializing the Mastermind software. RT has been employed by Genomenon, Inc.

## Footnotes

1 http://dgv.tcag.ca/dgv/app/home

2 https://clinicalgenome.org/

3 https://decipher.sanger.ac.uk/

4 http://cnv.gtxlab.com/

5 https://www.ncbi.nlm.nih.gov/pubmed

6 https://www.ncbi.nlm.nih.gov/books/NBK25497/

7 https://grobid.readthedocs.io/en/latest/

8 https://mastermind.genomenon.com/

9 https://www.genomenon.com/cvr/

10 https://www.ncbi.nlm.nih.gov/mesh

11 https://www.genenames.org/

12 https://www.uniprot.org/

13 https://www.concertgenetics.com/

14 https://www.ncbi.nlm.nih.gov/gtr/

15 https://www.google.com/

16 https://mastermind.genomenon.com/api

17 https://www.ncbi.nlm.nih.gov/snp/

18 https://varnomen.hgvs.org/

19 http://www.hgmd.cf.ac.uk/ac/index.php

20 http://clinvar.com/

21 https://scholar.google.com/

22 http://genome.ucsc.edu/cgi-bin/hgTables

23 https://www.ncbi.nlm.nih.gov/refseq/

24 https://www.illumina.com/products/by-type/clinical-research-products/trusight-rna-fusion.html

25 https://mastermind.genomenon.com/api

26 https://github.com/Genomenon/mastermind-api-cookbook

27 https://cancer.sanger.ac.uk/cosmic

## References

[B1] Amendola L. M. Jarvik G. P. Leo M. C. McLaughlin H. M. Akkari Y. Amaral M. D. (2016). Performance of ACMG-AMP variant-interpretation guidelines among nine laboratories in the clinical sequencing exploratory research consortium. *Am. J. Hum. Genet.* 99:247. 10.1016/j.ajhg.2016.06.001 27392081 PMC5005465

[B2] Birgmeier J. Deisseroth C. Hayward L. Galhardo L. Tierno A. Jagadeesh K. (2020). AVADA: towards automated pathogenic variant evidence retrieval directly from the full text literature. *Genet. Med.* 22 362–370. 10.1038/s41436-019-0643-6 31467448 PMC7301356

[B3] Catalogue of Somatic Mutations in Cancer [COSMIC] (2020). *Gene Fusions in Cancer.* Available at: https://cancer.sanger.ac.uk/cosmic/fusion (accessed May 2020). 10.1002/0471142905.hg1011s57 PMC2705836 18428421

[B4] The Center for Applied Genomics (2020). *Database of Genomic Variants - A Curated Catalogue of Human Genomic Structural Variation.* Available at: http://dgv.tcag.ca/dgv/app/home (accessed July 2020).

[B5] ClinGen - Clinical Genome Resource (2020). Available at: https://clinicalgenome.org/.

[B6] CNVdigest (2020). *CNVdigest.* Available at: http://cnv.gtxlab.com/ (accessed July 2020).

[B7] Concert Genetics (2020). *Connecting the Genetic Health Information Network | Concert Genetics.* Available at: https://www.concertgenetics.com/ (accessed October 2018).

[B8] Cooper D. N. Ball E. V. Krawczak M. (1998). The human gene mutation database. *Nucleic Acids Res.* 26 285–287. 10.1093/nar/26.1.285 9399854 PMC147254

[B9] DECIPHER (2020). *DECIPHER: Database of Chromosomal Imbalance and Phenotype in Humans using Ensembl Resources.* Available at: https://decipher.sanger.ac.uk/ (accessed July 2020). 10.1016/j.ajhg.2009.03.010 PMC2667985 19344873

[B10] Gao Q. Liang W. W. Foltz S. M. Mutharasu G. Jayasinghe R. G. Cao S. (2018). Driver fusions and their implications in the development and treatment of human cancers. *Cell Rep.* 23 227–238e223. 10.1016/j.celrep.2018.03.050 29617662 PMC5916809

[B11] Genomenon, Inc (2020a). *Mastermind - Comprehensive Genomic Search Engine.* Available at: https://mastermind.genomenon.com/.

[B12] Genomenon, Inc (2020b). *Mastermind Cited Variants Reference.* Available at: https://www.genomenon.com/cvr/ (accessed July 2020).

[B13] Genomenon, Inc (2020c). *Mastermind API.* Available at: https://mastermind.genomenon.com/api (accessed July 2020).

[B14] gnomAD (2020). *Downloads | gnomAD.* Available at: https://gnomad.broadinstitute.org/downloads (accessed July 2020).

[B15] Google (2020a). *Google.* Available at: https://www.google.com/ (accessed October 2018).

[B16] Google (2020b). *Google Scholar.* Available at: https://scholar.google.com/ (accessed October 2019).

[B17] Gradishar W. Johnson K. Brown K. Mundt E. Manley S. (2017). Clinical variant classification: a comparison of public databases and a commercial testing laboratory. *Oncologist* 22 797–803. 10.1634/theoncologist.2016-0431 28408614 PMC5507641

[B18] Gu Z. Churchman M. L. Roberts K. G. Moore I. Zhou X. Nakitandwe J. (2019). PAX5-driven subtypes of B-progenitor acute lymphoblastic leukemia. *Nat. Genet.* 51 296–307. 10.1038/s41588-018-0315-5 30643249 PMC6525306

[B19] Harrison S. M. Dolinsky J. S. Knight Johnson A. E. Pesaran T. Azzariti D. R. Bale S. (2017). Clinical laboratories collaborate to resolve differences in variant interpretations submitted to ClinVar. *Genet. Med.* 19 1096–1104. 10.1038/gim.2017.14 28301460 PMC5600649

[B20] Hoskinson D. C. Dubuc A. M. Mason-Suares H. (2017). The current state of clinical interpretation of sequence variants. *Curr. Opin. Genet. Dev.* 42 33–39. 10.1016/j.gde.2017.01.001 28157586 PMC5446800

[B21] Hugo Gene Nomenclature Committee [HGNC] (2020). *Home | HUGO Gene Nomenclature Committee.* Available at: https://www.genenames.org/ (accessed July 2020).

[B22] Human Genome Variation Society [HGVS] (2020). *Sequence Variant Nomenclature.* Available at: https://varnomen.hgvs.org/ (accessed July 2020).

[B23] Illumina (2020). *TruSight RNA Fusion Panel | Fusion detection in cancer research samples.* Available at: https://www.illumina.com/products/by-type/clinical-research-products/trusight-rna-fusion.html (accessed May 2020).

[B24] Landrum M. J. Lee J. M. Benson M. Brown G. R. Chao C. Chitipiralla S. (2018). ClinVar: improving access to variant interpretations and supporting evidence. *Nucl. Acids Res.* 46 D1062–D1067. 10.1093/nar/gkx1153 29165669 PMC5753237

[B25] Li Q. Wang K. (2017). InterVar: clinical interpretation of genetic variants by the 2015 ACMG-AMP guidelines. *Am. J. Hum. Genet.* 100 267–280. 10.1016/j.ajhg.2017.01.004 28132688 PMC5294755

[B26] Lopez P. (2020). *GROBID Documentation.* Available at: https://grobid.readthedocs.io/en/latest/ (accessed July 2020).

[B27] Mitelman F. Johansson B. Mertens F. (2007). The impact of translocations and gene fusions on cancer causation. *Nat. Rev. Cancer* 7 233–245. 10.1038/nrc2091 17361217

[B28] National Center for Biotechnology Information [NCBI] (2020a). *PubMed.* Available at: https://www.ncbi.nlm.nih.gov/pubmed/ (accessed October 2019).

[B29] National Center for Biotechnology Information [NCBI] (2020b). *ClinVar.* Available at: http://clinvar.com/ (accessed October 2019).

[B30] National Center for Biotechnology Information [NCBI] (2020c). *Home - Genetic Testing Registry (GTR) - NCBI.* Available at: https://www.ncbi.nlm.nih.gov/gtr/ (accessed October 2018).

[B31] National Center for Biotechnology Information [NCBI] (2020d). *Home - MeSH - NCBI.* Available at: https://www.ncbi.nlm.nih.gov/mesh/ (accessed July 2020).

[B32] National Center for Biotechnology Information [NCBI] (2020e). *Home - SNP - NCBI.* Available at: https://www.ncbi.nlm.nih.gov/snp/ (accessed July 2020).

[B33] National Center for Biotechnology Information [NCBI] (2020f). *RefSeq: NCBI Reference Sequence Database.* Available at: https://www.ncbi.nlm.nih.gov/refseq/ (accessed July 2020).

[B34] Ng P. C. Henikoff S. (2001). Predicting deleterious amino acid substitutions. *Genome Res.* 11 863–874. 10.1101/gr.176601 11337480 PMC311071

[B35] Online Mendelian Inheritance in Man [OMIM] (2020). *An Online Catalogue Of Human Genes And Genetic Disorders.* Available at: https://www.omim.org/ (accessed July 2020).

[B36] Orphanet (2020). *The Portal for Rare Diseases and Orphan Drugs.* Available at: https://www.orpha.net/consor/cgi-bin/index.php (accessed July 2020). PMC3669585 23761721

[B37] Pareja F. Lee J. Y. Brown D. N. Piscuoglio S. Gularte-Merida R. Selenica P. (2019). The Genomic Landscape of Mucinous Breast Cancer. *J. Natl. Cancer Inst.* 111 737–741. 10.1093/jnci/djy216 30649385 PMC6624163

[B38] PolyPhen-2 (2020). *Prediction of Functional Effects Of Human nsSNPs.* Available at: http://genetics.bwh.harvard.edu/pph2/ (accessed July 2020).

[B39] Qiu F. Xu Y. Li K. Li Z. Liu Y. DuanMu H. (2012). CNVD: text-mining based copy number variation in disease database. *Hum. Mutat.* 33 E2375–E2381. 22826268 10.1002/humu.22163

[B40] Rai K. Pilarski R. Boru G. Rehman M. Saqr A. H. Massengill J. B. (2017). Germline BAP1 alterations in familial uveal melanoma. *Genes Chromos. Cancer* 56 168–174. 10.1002/gcc.22424 27718540 PMC5490375

[B41] Richards S. Aziz N. Bale S. Bick D. Das S. Gastier-Foster J. (2015). Standards and guidelines for the interpretation of sequence variants: a joint consensus recommendation of the American college of medical genetics and genomics and the association for molecular pathology. *Genet. Med.* 17 405–424. 10.1038/gim.2015.30 25741868 PMC4544753

[B42] Sayers E. (2010). *A General Introduction to the E-utilities.* Available at: https://www.ncbi.nlm.nih.gov/books/NBK25497/

[B43] Schwarz J. M. Rödelsperger C. Schuelke M. Seelow D. (2010). MutationTaster evaluates disease-causing potential of sequence alterations. *Nat. Methods* 7 575–576. 10.1038/nmeth0810-575 20676075

[B44] Tallegas M. Fraitag S. Binet A. Orbach D. Jourdain A. Reynaud S. (2019). Novel KHDRBS1-NTRK3 rearrangement in a congenital pediatric CD34-positive skin tumor: a case report. *Virchows Arch.* 474 111–115. 10.1007/s00428-018-2415-0 30187166

[B45] Tate J. G. Bamford S. Jubb H. C. Sondka Z. Beare D. M. Bindal N. (2019). COSMIC: the catalogue of somatic mutations in cancer. *Nucl. Acids Res.* 47 D941–D947. 10.1093/nar/gky1015 30371878 PMC6323903

[B46] Tomczak K. Czerwinska P. Wiznerowicz M. (2015). The Cancer Genome Atlas (TCGA): an immeasurable source of knowledge. *Contemp. Oncol.* 19 A68–A77. 10.5114/wo.2014.47136 25691825 PMC4322527

[B47] Turner J. A. Bemis J. G. T. Bagby S. M. Capasso A. Yacob B. W. Chimed T. S. (2019). BRAF fusions identified in melanomas have variable treatment responses and phenotypes. *Oncogene* 38 1296–1308. 10.1038/s41388-018-0514-7 30254212

[B48] UniProt (2020). *UniProt.* Available at: https://www.uniprot.org/ (accessed July 2020).

[B49] University of California, Santa Cruz (2020). *UCSC Genome Browser - Table Browser.* Available at: http://genome.ucsc.edu/cgi-bin/hgTables (accessed July 2020).

[B50] Velusamy T. Kiel M. J. Sahasrabuddhe A. A. Rolland D. Dixon C. A. Bailey N. G. (2014). A novel recurrent NPM1-TYK2 gene fusion in cutaneous CD30-positive lymphoproliferative disorders. *Blood* 124 3768–3771. 10.1182/blood-2014-07-588434 25349176

[B51] Yen J. L. Garcia S. Montana A. Harris J. Chervitz S. Morra M. (2017). A variant by any name: quantifying annotation discordance across tools and clinical databases. *Genome Med.* 9:7. 10.1186/s13073-016-0396-7 28122645 PMC5267466

[B52] Zehir A. Benayed R. Shah R. H. Syed A. Middha S. Kim H. R. (2017). Mutational landscape of metastatic cancer revealed from prospective clinical sequencing of 10,000 patients. *Nat. Med.* 23 703–713. 10.1038/nm.4333 28481359 PMC5461196

